# Reflections on Inner and Outer Silence and Consciousness Without Contents According to the Sphere Model of Consciousness

**DOI:** 10.3389/fpsyg.2020.01807

**Published:** 2020-08-12

**Authors:** Patrizio Paoletti, Tal Dotan Ben-Soussan

**Affiliations:** Research Institute for Neuroscience, Education and Didactics, Patrizio Paoletti Foundation, Assisi, Italy

**Keywords:** consciousness without content, Sphere Model of Consciousness, silence, default mode network, insula

## Abstract

In the current hypothesis paper, we propose that focusing attention on silence can be used as a paradigm conceptually similar to sensory deprivation, to study consciousness without content. We briefly overview recent influential models of consciousness and consider how they assess the relationship between consciousness and contents. After discussing the strengths and weaknesses of current models, we suggest an extension based on the Sphere Model of Consciousness (SMC) and introduce new definitions for identification and self-awareness as states of consciousness. We further compare Paoletti’s theoretical model for the development of self with other influential models, highlighting similarities and differences. We conclude with a discussion of how attentional focus on silence can be empirically tested.

## Introduction

Several recent theoretical models have aimed to clarify the idea of consciousness in itself, independent of its contents. Examples include the examination of *metacognitive consciousness* (Raffone and Srinivasan, 2009), consciousness with minimal content (Metzinger, 2018), and *consciousness-as-such* (Josipovic, 2019). In this context, we recently presented the Sphere Model of Consciousness (SMC; Paoletti, 2002a,b, 2008; Paoletti and Selvaggio, 2011; Paoletti and Ben Soussan, 2019), which attempts to represent the phenomenology of consciousness using the geometrical properties of the sphere. In the present hypothesis paper, we propose that focusing attention on silence can be used as a paradigm similar to sensory deprivation, to study consciousness without content. Our hypotheses, elaborated below, are based on the SMC.

Our hypothesis, that attention to silence creates a paradigm similar to sensory deprivation, is based on a specific notion of environment. The concept of environment can be defined in various ways. Here, we refer to both internal and external aspects of the environment, as follows: the internal environment encompasses intrinsic determinants, namely the person’s psychological, neurological, and physiological mechanisms, while the external environment comprises extrinsic determinants, such as perceptual deprivation (Glicksohn, 1991; Ben-Soussan et al., 2019). Internal and external environments do not act independently on the individual but rather work together to shape thoughts, feelings, and behaviors (De Fano et al., 2019). More specifically, we will explore the difference between internal intentional acts, such as meditation, and external perceptual deprivation, keeping in mind that structured external environments can also facilitate better internal communication (Paoletti and Selvaggio, 2013; Paoletti et al., 2017). We aim to explore the difference between internal intentional acts and external deprivation, using silence as an example of an intentionally achieved internal environment supporting a state of consciousness-as-such or consciousness without content.

We begin with a brief review of perspectives on the relationship between consciousness and content, as presented in recent studies. After considering the strengths and weaknesses of current models, specifically in relation to the isolation of consciousness from its contents, we suggest an extension based on the SMC. As suggested by one of our reviewer, these definitions are briefly compared with those used in other hierarchical models, including Maslow’s hierarchy of needs (Maslow and Lewis, 1987), Wilber’s Integral Theory (Wilber, 1979, 2000), and Drigas and Pappas’ Consciousness-Intelligence-Knowledge Pyramid (Drigas and Pappas, 2017), as well Paoletti’s theoretical model of the development of the self (Paoletti et al. 2016). We further introduce new definitions for *identification* and *self-awareness* as states of consciousness.

## Differentiating Between Consciousness and its Content

### How Current Models Address the Differentiation Between Consciousness and Content

Three of the most recent models of consciousness, proposed by Raffone and Srinivasan (2009), Metzinger (2018), and Josipovic (2019), vary in their views on the content of consciousness and on how it can be differentiated from consciousness itself.

Raffone and Srinivasan (2009) suggested that access to consciousness is determined by selective endogenous attention, which produces a “working access bias” to the stimuli relevant for the response (target) in a given situation and consequent stimulus-response mapping. They also proposed that several dynamic links in the adaptive coding networks [e.g., in the dorsolateral prefrontal cortex, anterior prefrontal cortex, and anterior cingulate cortex (ACC)] could connect to enable access to consciousness. This processing of information is then modulated by body state and environment. Raffone and Srinivasan further proposed a classification of three orders of consciousness processes in relation to perception. With Baars’s Global Workspace Theory^1^ (GWT; Baars, 1983, 1998, 2002; Baars et al., 2003) as a starting point, they suggested that in the first order of consciousness processes, each cluster of neurons which constitutes a core of the global workspace (GW) in the adaptive coding networks relates to a single perceptual object, while a larger set of backstage neurons operating at the unconscious level support and modulate conscious perception. Thus, phenomenal consciousness of a given object is implicit or contextual, while the specific characteristics of the object of consciousness are mediated by the cores of the GW. When the cores dedicated to the specific object decays, the previously backstage networks supporting it emerge in consciousness, resulting in first person phenomenal awareness of subjective states perceived as “I.” This second order contains the introspective experiences of self-perception. This “I” will then be considered simultaneously in relation to body states and the environment, through the production of neuronal connections between adaptive networks and neural markers of transient body states. Finally, the third order of consciousness refers to the possibility that adaptive neuronal networks act non-referentially, that is, only recursively as consciousness of being conscious.

Raffone and Srinvasan (2009) also argue that in metacognitive processes, alpha coherence^2^ has a decisive role in guiding the dynamics of neuronal populations with adaptive coding properties in the prefrontal cortex. Based on the above, they conclude that “awareness of being aware” can be understood only as an “intuitive awareness” based on meditation (Sumedho, 2004). While Raffone and Srinivasan provide an insightful depiction of consciousness dynamics referred to in Open Monitoring (OMM) and Focused Attention Meditation (FAM), they note that the same processes are potentially observable in a wide range of cognitive settings and experiential contexts other than meditation. As such, Raffone and Srinivasan’s model raises challenges in isolating contents from consciousness. More specifically, if metacognitive awareness is a process of “intuitive awareness” underlying other processes observable solely by expert meditation practitioners, and in a wide range of settings and contexts, it is difficult to isolate metacognitive processes from contents in consciousness.

Metzinger’s *Ascending Reticular Arousal System* (ARAS) model (Metzinger, 2018) differs considerably from that of Raffone and Srinivasan (2009), addressing what he calls Minimal Phenomenal Experience (MPE). In Metzinger’s view, the MPE models the global state of arousal in the brain. According to this view, consciousness-as-such must be related to a mechanism underlying any kind of content, and it is believed to be the general state of arousal in the brain. The general state of arousal appears as a representation when its reflexivity is not activated and appears as non-representational when its reflexivity is activated. The state of arousal becomes the signature of consciousness when its reflexivity is activated, and the individual perceives it in a state of “content-less wakefulness,” like it occurs in lucid dreams. This model bonds consciousness without contents to level of arousal with tautology as a potential side effect: that is to say that consciousness requires arousal and arousal can be a content in itself for the brain (Josipovic, 2019).

Finally, according to Josipovic (2019), consciousness-as-such or nondual awareness is viewed as the foundation of consciousness, independent of any other phenomenal content: an empty awareness that is non-conceptual and without subject-object structuring. In Josipovic’s view, determining roles are played by a dynamic functional network with a main node in the central area of the precuneus and a main axis with a node in the dorso-lateral prefrontal cortex. Josipovic notes that the precuneus is the most connected hub in the cortex, involved in perceptual, motor, affective, and cognitive functions. *Nondual awareness* is believed to function as a background framework that, when included in the GW, unifies contents. Thus, Josipovic hypothesizes that the neural distinction between *consciousness-as-such* and content is that neural activity related to the former is reduced to precuneus and neighboring areas when *nondual awareness* is isolated from all contents, accompanied by synchronized activity within low gamma (40–60 Hz) or high gamma (above 100 Hz) ranges. Finally, Josipovic emphasizes that the signature of consciousness-as-such lies not in the difference between conscious and unconscious contents but in dynamic brain patterns common to both. Josipovic’s model also delineates a specified research protocol addressing changes in the dynamics and scope of the central precuneus network, aiming to specify the neuronal population related to “nondual awareness” and, more specifically, to its different dimensions (see Josipovic, 2019 for further detail).

The three models summarized above provide detailed frameworks for investigating the correlates of consciousness in itself, but there is room to address additional variables in empirical research, such as settings thought to observe differences, for example, between expert and non-expert meditation practitioners and between conditions intentionally sought or outwardly imposed. Both conscious and unconscious experiential contents are thought to be generated through mutual interactions between perceptual, affective, and cognitive processes (Metcalfe and Son, 2012; Vandekerckhove et al., 2014; Barrett, 2017; Josipovic, 2019). We can explore the possibility of setting constraints on the three types of processes and investigating each one separately to learn about its relationship with consciousness. We begin below with the perceptual level, proposing that perceptual deprivation (here defined as a homogeneous perceptual field) can be used to isolate consciousness in itself from contents. We then discuss silence as a means of achieving perceptual deprivation.

### Perceptual Deprivation (or Saturation) as a Means of Isolating Consciousness in Itself From Contents

The concepts of absorption and transcendence are by no means new; they have been discussed in the philosophies of both Eastern and Western traditions (e.g., Odin, 1981; Solomon, 1985, for reviews) and investigated scientifically for over 40 years. More specifically, perceptual deprivation has long been used to elicit the state of absorption (Tellegen and Atkinson, 1974). Notwithstanding ambiguity associated with research on absorption (Roche and McConkey, 1990), it has proven beneficial in recent research using mindfulness to study altered states of consciousness (Lau et al., 2006).

It has been proposed that absorption, as experienced by meditation practitioners, can be studied as a means of differentiating consciousness in itself from (other) phenomenal contents (Josipovic, 2014). In this regard, absorption is considered both a trait and a state (Ben-Soussan et al., 2019). As a trait, absorption involves the ability to highly focus attention (Tellegen and Atkinson, 1974) and is related to meditation, empathy, and hypnotic ability (Lau et al., 2006). State of absorption has been assessed using questions like “I was not distracted but was able to become completely absorbed in what I was experiencing” (Hall et al., 2016).

Individuals who score highly on trait absorption, an “inherently interactive” trait (Tellegen, 1981), tend to experience states of absorption given suitable circumstances (Glicksohn, 1987, 2004). Suitable circumstances are, for instance, altered immersive sensory environments, such as sensory homogenization (e.g., ganzfeld, or any visual condition without temporal or spatial contrast, like an unclouded sky; Baars, 2013). These immersive sensory environments facilitate the induction of altered states of consciousness (Glicksohn, 1991) similar to meditation (Benson, 2001; Lindahl et al., 2014). As such, exposure to an immersive environment, such as whole-body perceptual deprivation, should elicit experiences of absorption (Glicksohn and Berkovich-Ohana, 2012).

Considering the immersive nature of such altered environments, we propose the term perceptual *saturation*, rather than *deprivation*. Saturation could be a more fitting naming for what has been called *sensory overload* (Vollenwieder and Geyer, 2001). In fact, also Vaitl et al. (2005) defined these types of environments as *restricted stimulation*. Over the years, a correlation between sensory overload and difficulty in distinguishing self from non-self in both healthy altered states of consciousness and psychopathological cases has been reported (Scharfetter, 1981; Vollenweider, 1998; Vollenwieder and Geyer, 2001; Jamieson, 2007).

Glicksohn et al. (2017) and Ben-Soussan et al. (2019) conducted a series of studies with the OVO Whole-Body Perceptual Deprivation (OVO-WBPD) chamber, which is an altered sensory environment in the form of a human-sized egg (*Uovo* in Italian literally means egg). Based on the SMC, detailed below, the OVO-WBPD was specifically built with the aim of facilitating an immersive experience and an increased state of presence (Paoletti, 2002a). The electrophysiological fingerprint of the transition into an absorption state induced by the OVO-WBPD chamber was enhanced delta and beta activity, left lateralized to the inferior frontal region, peaking at the insula (Ben-Soussan et al., 2019). The insula plays a role in the experience of bodily self-awareness, sense of action, and sense of body possession (Craig, 2009), in addition to transmitting homeostatic information that enables sensory integration (Williamson et al., 2001; Xue et al., 2010). As such, these results suggest enhanced effort to regulate the embodied self, based on interoception, when multi-sensory integration normally provided by the external visual field is impeded. The involvement of the insula suggests that absorption can be experienced when information simultaneously saturates the whole spectrum of perception, due to the immersive homogenous environment.

We can interpret the OVO-WBPD experiment as a constraint on content creation at the perceptual level. Constraining a specific level in this manner might allow us to observe, in greater detail, the process of achieving consciousness without contents. In the following section, we propose that silence can be used as a kind of perceptual saturation, intentionally sought by meditation practitioners.

## Silence As “Perceptual Saturation”

Silence is frequently associated with contemplative practices and meditation, but it is not often discussed in neuroscientific studies on the neuronal effects of meditation. This may be due to the fact that silence, as opposed to noise, is difficult to define in positive terms. Indeed, silence is usually discussed in the scientific literature with reference to its absence, as in investigations of noise pollution damage (Wagner et al., 2004; WHO, 2011). Such research has shown that excessive noise has a direct negative impact on learning (Sanz et al., 1993; Wagner et al., 2004; WHO, 2011) and produces sleep disturbances with negative effects on insight production (Wagner et al., 2004).

Silence can be thought of as non-phenomenal or as an empty phenomenon. For example, when Radin et al. (2011) investigated attention, they used silence as a negative indicator, looking at how sound led meditation practitioners to distraction. In this sense, the construct of silence is comparable to that of consciousness without content: silence is identified only in contrast to its interruption, just as consciousness is considered only in terms of the contents that cancel its vacuity.

A method used in many traditional practices, called *listening to silence* (Teschner, 1981; Davies and Turner, 2002; Stratton, 2015), allows us to consider this construct from a different perspective. We can then ask which cognitive and/or physiological resources are required to draw attention to something that seemingly cannot be the object of attention, or cannot be defined, and hypothesize that those resources are similar to those involved in sensory saturation.

Neurally speaking, silence could work like an immersive sensory environment, but one that is intentionally sought by the practitioner. The importance of intentionality here is central because sensory and perceptual deprivation has been reported as a positive or aversive experience (Hebb, 1955; Feinstein et al., 2018; Ben-Soussan et al., 2019) depending on its intention. For example, while isolating participants against their own will can be considered white torture (Brown, 2007; Mausfeld, 2009), over the centuries, people have voluntarily utilized isolation as means of getting reconnected to themselves and achieve higher states of consciousness (Ustinova, 2009, 2017). This could mean that intention, or at least the perception of self-determining the experience, namely the way one choses to interpret the event and voluntarily orient it to his/her aims, has a decisive role in the experience of sensory deprivation. In the context of meditative traditions centered on “listening to silence” (Teschner, 1981; Davies and Turner, 2002; Stratton, 2015), we consider more the intentional use of silence, than only physical silence. Particularly relevant in this context is the work of Scholl et al. (2010), indicating that a special neuronal network is dedicated to silence. Accordingly, like the immersive sensory environment, silence could elicit an observable process involving a hypothesized network dedicated to listening to silence with noticeable differences between external silent sensory immersive environments and intentionally sought silence. Indeed, according to the SMC, intentionality is a decisive factor in reaching a state of consciousness without content.

To advance our exploration of the neural mechanisms underlying attention to silence, considered here as a kind of sensory saturation that challenges the brain to integrate perceptions, we present a phenomenological matrix that aims to represent consciousness through the SMC (Paoletti, 2002a,b, 2008; Paoletti and Ben-Soussan, 2019). The sphere represents a matrix in which the experiences of consciousness can be effectively placed both statically and dynamically. It is therefore a good basis for describing, phenomenologically, the consciousness-related experiences of contemplative practice and meditation practitioners. Most importantly, the geometric form of the sphere is suitable for describing consciousness without content, taking into account the characteristics of phenomenal experiences. After presenting the SMC, we use it to elaborate on the specific relationships between silence and consciousness without content.

## The Sphere Model of Consciousness

The description of consciousness within the space of a matrix is based on the *space-state* concept (Fell, 2004; Werner, 2009; Berkovich-Ohana and Glicksohn, 2014), which has been employed to describe consciousness as a system and define its dimensions and dynamics. In the current paper, we adopt the SMC as a framework.

### The Sphere as an Icon of Consciousness

**Figure 1:**
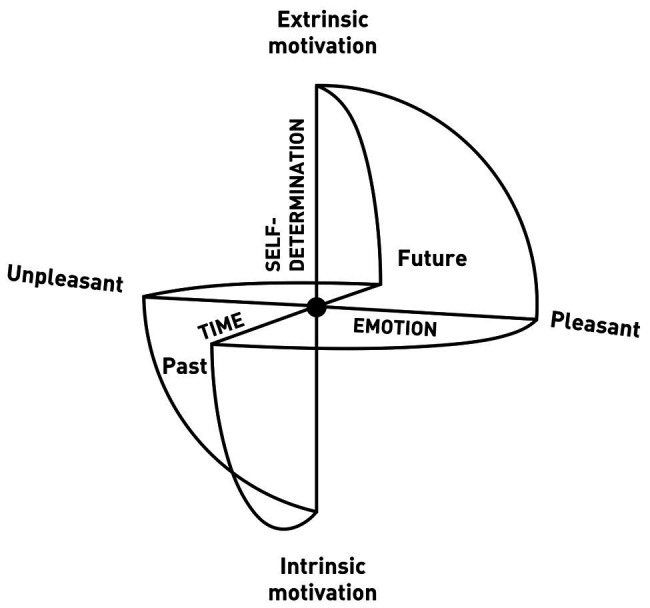
The Sphere Model of Consciousness (SMC), axes, and polarities. Adapted from Paoletti and Ben-Soussan (2019).

In describing consciousness without content, in a certain sense, we are faced with the need to represent the unrepresentable or express the inexpressible. A primary feature of the sphere is its emptiness. The classic geometrical definition describes it as a set of points equidistant from a center (Hilbert et al., 1952), but if we open up a ping pong or tennis ball, we see that this center is invisible. This invisible center can be conceptualized as the heart of consciousness, equidistant from all stressors (see Figure 1).

Recalling the discussion of immersive environments and absorption above, we can hypothesize that when consciousness, through attention, expands and becomes able to be in connection with all contents without any of them prevail, the contents vanish due to lack of differentiation, as in the case of the ganzfeld homogeneous perceptual field^3^ (Wackermann et al., 2002; Miskovic et al., 2019). This, in turn, leads to the experience of “loss of self” (Avant, 1965). When consciousness is guided by expanded attention due to sensory saturation (e.g., perceptual deprivation; Leckart et al., 1970) to the point of dissolving identification with specific content and even the distinction between self and non-self (Vollenwieder and Geyer, 2001), the specific content vanishes, and the subject could enter a state of consciousness without contents. As we saw, sensory deprivation or saturation could be experienced in opposite ways, i.e., as white torture (Brown, 2007; Mausfeld, 2009), or to achieve higher states of consciousness (Vaitl et al., 2005; Ustinova, 2009, 2017). According to the SMC, the opportunity to enter a state of consciousness without content through sensory saturation is assumed to be determined by intentionality, represented in the model with the vertical axis (see Figure 1). This could mean that intentionality is a necessary element for experiencing consciousness without content. In the SMC, when the consciousness is intentionally expanded to all contents in a state of non-attachment, the center of the sphere, which represents the meeting point of the three axes, becomes what we have called the *place of pre-existence* (Paoletti and Ben-Soussan, 2019). This name is based on the hypothesis that, in the central place, when there is intentional non-identification with any content, perception can be experienced without the usual filters created by memories. This makes it possible to reprocess subjective autobiographical memories and implies that we can emerge from a state of absorption with a more neutral relationship with our own memories, without losing them. Accordingly, Vaitl et al. (2005) observed that after participation in a restricted stimulation compared with rest condition, autobiographical life episodes were retrieved more intensely and recalled more pleasantly.

The term place of pre-existence indicates the receptive and originally undifferentiated character of consciousness, with respect to all stimuli within and outside the individual. This is the geometrical place in the matrix where we situate the possibility of consciousness-as-such, which we parallel here with silence. In the model, the three ideal axes represent three lines of force passing through the central point, such that the intersection divides the axes into two sections (Figure 1).

As a mathematical metaphor, the sphere constitutes an ideal paradigmatic model. One of its fundamental characteristics is that all the points are “umbilical,” or locally spherical. This means that the normal curvature is the same in all directions, and each tangent vector represents a main direction. Hence, the logic of the sphere is a logic of equitable redistribution of forces (Hilbert et al., 1952). Every pressure that reaches the sphere from one of the six directions of space, which in the model represent the polarizations of time, emotion, and self-determination, is redistributed on the whole structure without creating deformations. This continues provided that the center of the sphere remains “empty,” or that consciousness is not drawn back to identification with specific content.

### The Center of the Sphere

**Figure 2:**
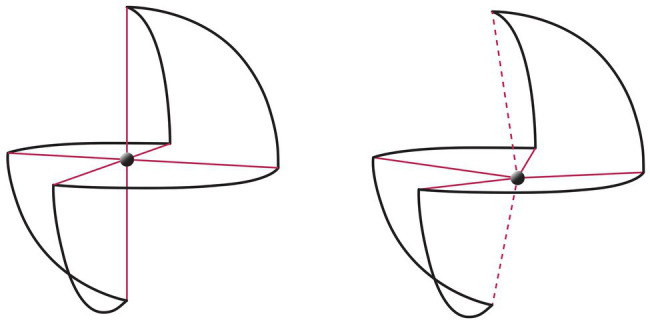
**(A)** The centered condition, in which all three axes intersect at the center of the sphere. **(B)** A decentered condition, in which the intersection is only between the time and emotion axes, and there is not intentional self-determination. The dotted lines depict the state of no voluntary activation of the self-determination axis.

To fully understand the function of the center of the geometrical matrix of the SMC, it is necessary to note that the model is (at least) three-dimensional (3D) and dynamic, and that the intersection point between the axes represents the phenomenological coordinates of experience at a given moment. If self-determination (third axis) does not participate, the intersection will be defined only by the first two axes, time, and emotion. When there is no voluntary activation of the self-determination axis, the intersection point can be “decentralized.” Thus, one is conditioned by memories and feeling; or, in other words, those things that capture your memories and feelings determine who you are. Visually, when the experience of consciousness is characterized by identification with one of the polarities of time and emotion, this can be represented as a shift of the center point and consequent deformation of the axes. For example, if a practitioner’s mind is projected to a future unpleasant experience, it would be placed in the model in an intermediate point between the future unpolarity and pleasant polarity (see Figure 2).

**Figure 3:**
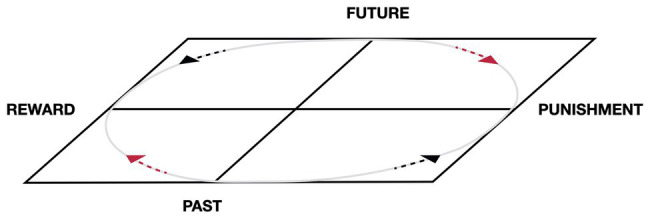
The “flat life,” the intersection of the two axes—time and emotion—produces a two-dimensional (2D) and circular life, or a “flat life,” in which the repetition of experience cycles is determined by the interaction between emotional memories and projective experiences; that is, a circuit that moves between dependence and the need for gratification. Adapted from Paoletti and Ben-Soussan (2019).

When the characteristics of the state of consciousness produced by the relation between time and emotion are in equilibrium with each other, but the dimension of self-determination is not intentionally manifested, we will have a graphic localization in the center of the sphere. This is referred to in the model as “flat life” or “bidimensional consciousness” (see Figure 3, Paoletti and Ben-Soussan, 2019). The center, in this case, represents the intermediate point between the axes, but it does not yet represent the non-dual state of consciousness (Josipovic, 2014; Vieten et al., 2018) discussed in “Silence and Consciousness without Content” section. For example, if the state is characterized by equilibrium, but the equilibrium is not reached intentionally, the center will represent the present moment and/or the emotional balance but not the non-dual state. Any experience of the present implies attention toward some references, and the references are placeable along the axes. Only when the axis of self-determination is inserted and a balance is created between all the polarities of experience will the subject experiences a non-dual awareness. Thus, we can have a two-dimensional (2D) center, which is not necessarily the same as the 3D center that represents the place of pre-existence. The state at the center of the sphere, when it is experienced as the place of preexistence, would be not characterized by the absence of contents, but by the equidistant presence of all contents, with none prevailing in its relationship with self-perception.

**Figure 4:**
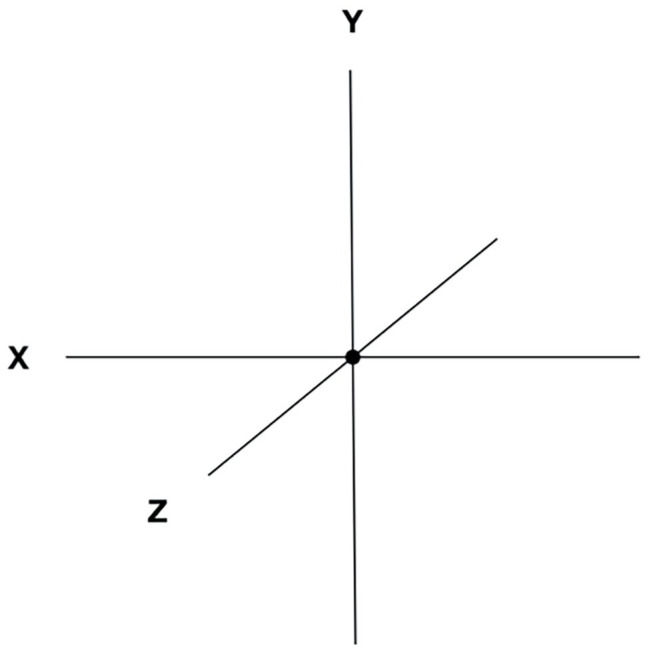
The three ideal axes represent three lines of force passing through the central ideal point, such that the intersection divides each axis into two sections.

### The Axes

Each axis in the SMC represents the unfolding and polarity of an aspect of experience, with an equilibrium point in the center of the sphere, and a graduated scale indicating distance from the center (Figure 4). With respect to the spatial coordinates, the back-front axis, called the *time axis*, represents the temporal unfolding of past to future; the right-left axis, *emotion axis*, represents the emotional polarities of unpleasant and pleasant; and the vertical axis, *awareness-self-determination axis*, represents the dimensions of value and aspiration (see Figure 1). From a phenomenal point of view, the axes do not necessarily represent a linear continuum. For example, while we are used to thinking of past, present, and future on a linear continuum, from a psychological point of view, past and future are both projections from the central point representing the present time (Bar, 2011; Wittmann, 2015).

**Figure 5:**
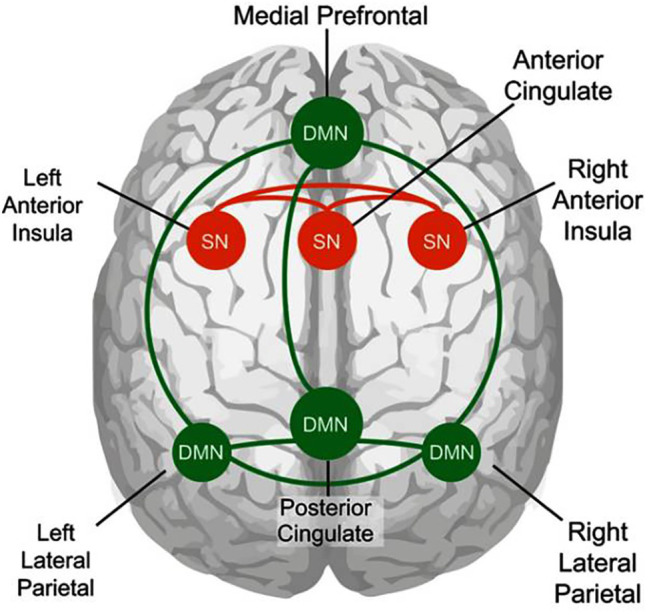
Node connections of the default mode network (DMN; shown in green) and the salience network (SN; shown in orange). Illustration of node connections used in this study. Adapted from van Ettinger-Veenstra et al. (2019).

The proposed correspondence between spatial coordinates and dimensions of experience is based on the assumption that sensorimotor circuits are the substrate of higher order cognitive processes (Rizzolatti and Sinigaglia, 2006). We can assume, therefore, that representations of space are the basis for more complex mental elaborations. As such, the SMC, as a matrix representing consciousness phenomena, is structured on the spatial directions, which parallel the coordinates of mental contents. The central point of the sphere represents the space of consciousness as characterized by the dimensions signified by the axes.

#### Time (Past-Future) and Emotion (Unpleasant-Pleasant) Axes

Autobiographical memory is composed of a stratification of perceptions, through molecular processes that have only been investigated partially (Bonhoeffer and Yuste, 2002; Malenka and Bear, 2004; Marie et al., 2005; Kim and Linden, 2007). Molecular research suggests that memory formation is modulated by several mood-related neurotransmitters, such as dopamine, serotonin, acetylcholine, and endocannabinoids (Marie et al., 2005). Parallel to the graphic representation of the SMC, these processes can be metaphorically represented by the encounter between the time and emotion axes.

We know, however, that memories necessarily record a subjective, partial view of experience, and that perception itself is constantly mediated by interpretation (Gazzaniga, 1985; Baars, 1998, 2002). This is supported by the temporal gap between sensory perception and consciousness of a stimulus, which in some cases can reach up to half a second (Libet, 1985; Dennet, 1989; Berry et al., 1999). We also know that memories are incessantly used by the brain for predictive activity, leading to the idea of the “proactive mind” (Bar, 2011), and that this activity influences sensory perception (Bjork, 1975; Schacter and Addis, 2007; Gazzaniga, 2012). Perception is a receptive rather than a passive process: if the stratification of memories constitutes the past in the SMC, projection represents the future (Farb et al., 2007; Wittmann, 2015). As an experience becomes more distant in time and space, it becomes increasingly abstract and subject to further elaboration. This greater level of abstraction comes with greater variability of interpretation (Johnson et al., 1988; Mather et al., 1997; Schacter and Slotnick, 2004; Loftus, 2005).

Turning to the emotion axis, many interpretative models proposed in the literature converge in claiming that emotional experience requires an omnipresent interpretative function, cognitive in nature. For example, according to Lambie and Marcel (2002), each emotional state is determined by the combination of two factors: readiness for action and evaluative description. Readiness for action refers to the ways in which brain and body systems are activated in response to stimuli. Evaluative description comprises self-representation of the ways one has been influenced by an event. This interpretative process, in the case of the emotions, exists through polarization. The emotions are pleasant or unpleasant and, from the psychological point of view, are associated with the reward/punishment dynamic and related brain networks (Carver and White, 1994; Delgado et al., 2000; Paoletti and Ben-Soussan, 2019).

The link between silence voluntarily reached through training, by traversing emotions and time, has been addressed by Vago and Zeidan (2016, p.13): “A sense of peace and quiet in the mind is proposed to arise through mental training in concentration, non-conceptuality, and discernment, in contrast to the untrained frenetic restlessness of mental time travel that is characteristic of daily activity in the postmodern setting.” The primary contribution of the current model is in the emphasis on the third axis, discussed below.

#### The Awareness-Self-Determination Axis

In the SMC, the ability to become self-determined is characterized with respect to two possible polarities, intrinsic motivation and extrinsic motivation (Deci, 1971; Moller et al., 2006; Paoletti and Ben-Soussan, 2019). At the two extremes of the axis are the concepts of *aspiration* (extrinsic motivation), conceptualized as the intentional pursuit of a pre-determined goal, and *values* (intrinsic motivation), conceptualized as “sacred values” (Berns et al., 2012) that the brain processes independently from the reward/punishment mechanism (Delgado et al., 2000). Aspiration denotes the most extreme form of extrinsic motivation and values the extreme form of intrinsic motivation.

Neuroimaging studies suggest that intrinsic motivation recruits the salience and central executive networks, while suppressing the default mode network (DMN; see Di Domenico and Ryan, 2017 for a recent review; Figure 5). The DMN is typically active during task-free resting states. It is thought to represent neural processing related to mind-wandering (Raichle et al., 2001; Buckner et al., 2008) and demonstrates decreased activity during effortful, goal-directed tasks (Greicius et al., 2003; Fox et al., 2005). The salience network, associated with the anterior insula (AI) and dorsal ACC, is believed to support the detection of subjectively important events and the mobilization of attentional and working memory resources in the service of goal-directed behavior (Menon and Uddin, 2010; Murayama et al., 2010; Menon, 2015). See Figure 3. For example, Murayama et al. (2015) found increased activity within the ACC and bilateral insula in response to free-choice cues, as compared to forced-choice cues. Regarding the comparison between intrinsic (such as “writing an enjoyable article”) and extrinsic motivation (e.g., “writing an extra-credit article”), increased activity within insular regions was found when participants imagined the enactment of intrinsically motivating activities (Lee et al., 2012; Lee and Reeve, 2013).

**Figure 6:**
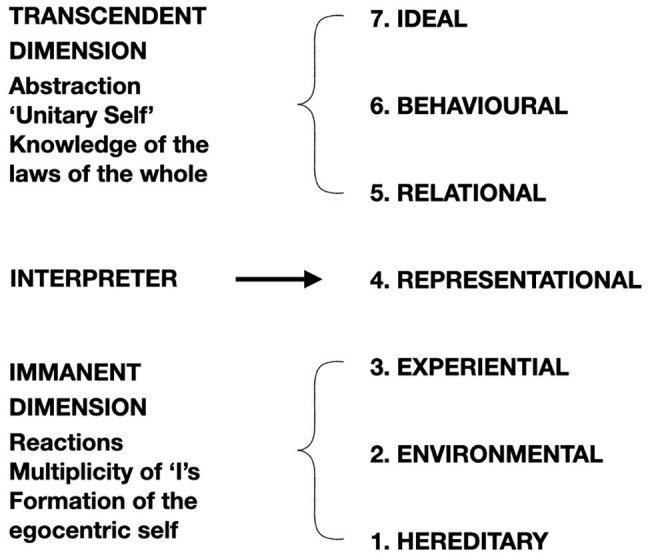
The theoretical model of development of the self, with the overall trisection and the seven levels of the self. Adapted from Paoletti et al. (2016).

Depraz et al. (2000) have described the subtle dynamic of “the act” of becoming more self-aware as necessarily intentional. They described three interdependent phases each of one characterized by intention: first, suspension from the habitual act of mind and body, then redirection of attention inwardly, and finally receptivity toward the experience. In the SMC, we hypothesize that self-determination is positively correlated with awareness (Paoletti and Ben-Soussan, 2019), as awareness improves adaptive decision-making, with positive effects on self-determination (Karoly, 1993). Regarding adaptivity and self-awareness, Seth and Baars (2005) claim that conscious events are highly informative, rapidly adaptive, internally coherent, and reportable. On this basis, they infer that a greater degree of consciousness corresponds to a better decision-making process, as every process of consciousness is in itself rich in information. We posit that there is a circular relationship between awareness and decision-making: while a greater degree of consciousness leads to better decision-making, it is also true that stronger motivation for decision-making improves awareness of the object of motivation. In this sense, awareness and self-determination are connected.

### Self-Awareness and Identification According to the Sphere Model of Consciousness

Having described the SMC in general, we can now discuss the significance of saturation in its context. An immersive environment, such as homogenization, can be interpreted as pressure equally exerted all around the sphere, pushing the self toward its center. In this respect, an “immersion” into silence is a means of saturation. The relationship between the center of the sphere and the axes is represented in the SMC by level of self-awareness, as explained below.

#### Minimal Self, Narrative Self, and Overcoming of the Self

**Figure 7:**
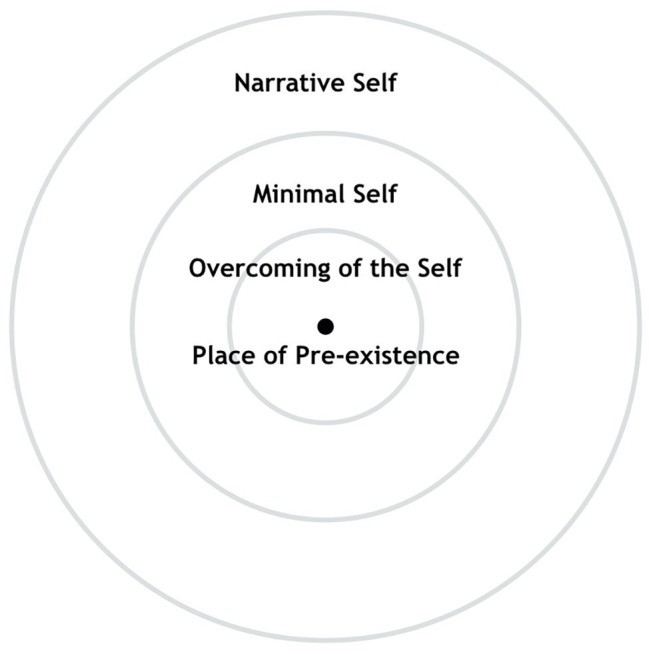
Minimal Self, Narrative Self, and Overcoming of the Self in the SMC. Adapted from Paoletti and Ben-Soussan (2019).

Neurophysiological research has often adopted the binary distinction between *Minimal self* and *Narrative self*. First proposed by James (1890/1950), the categories of Minimal and Narrative Selves were then redefined for the purposes of neuroscientific research by Gallagher (2000), with the former denoting the self as “I,” the knowing subject, a temporary presence, and the latter depicting the self as “me,” the object that is known, the concept of self and autobiographical identity. The Minimal Self has a short temporal extension and is endowed with a sense of action, property, and first person non-conceptual content, while the Narrative Self involves personal identity and continuity through time and includes conceptual content. In our previous papers on the SMC, we specified the addition of a third state, called Overcoming of the Self (see Figure 7, Paoletti and Ben-Soussan, 2019; Paoletti et al., 2020), in which all sense of self disappears. In the model, the Minimal Self is depicted as a circle close to the center, contained by a larger circumference that represents the Narrative Self.

We refer to the distinction between Minimal Self and Narrative Self because of its recurrent use in studies about neural correlates of the self. State and type of self are believed to have specific neural correlates. When the space of consciousness is completely occupied by contents placeable along the axes of the SMC, we have a state called “identification” with those contents. Neural activity in networks related to the Minimal Self or Narrative Self can indicate which kind of self-perception one is experiencing. Current research has indicated several neural correlates of the two types of self and suggested that they are hierarchically structured. Narrative Self is dependent on Minimal Self and not vice versa (for a review, see Berkovich-Ohana and Glicksohn, 2014). Meanwhile, Overcoming of the Self, which is parallel to consciousness without contents, has only recently been the subject of neuroscientific studies (Hinterberger et al., 2014; Winter et al., 2019). It can further be compared with self-transcendence, absorption, and non-dual states (Josipovic, 2014; Vieten et al., 2018), which are discussed in “Silence and Consciousness without Content” section.

To consider the SMC in the context of other hierarchical models, we compare it to Maslow’s hierarchy of needs (Maslow and Lewis, 1987), Wilber’s Integral Theory (Wilber, 1979, 2000), and Drigas and Pappas’ Consciousness-Intelligence-Knowledge Pyramid (Drigas and Pappas, 2017). As specified in our previous paper about the SMC (Paoletti and Ben-Soussan, 2019), in which we suggested neural correlates for the model, we limited our discussion to the concepts of Minimal and Narrative Selves and introduced the aforementioned dimension of Overcoming of the Self. Neural correlates for the two dimensions of Minimal Self and Narrative Self have been proposed and addressed in a vast body of literature. However, as suggested before, the complete SMC includes an evolutive representation for seven levels of the self (Paoletti et al., 2016; Paoletti and Ben-Soussan, 2019; Figure 6).

In the theoretical model of the development of the self, we have a tripartite structure that can be represented in the form of an hourglass, with the immanent dimension at the base and the transcendent dimension at the top. The central tipping point is the representational self. Different needs and aspirations also correspond to different levels of the self. At the base, in the hereditary self, the dimension of the basic needs is decisive (as in Maslow’s hierarchy); at the top, the ideal self is motivated by aspirations that transcend the individual and are oriented toward collectivity.

Once again, the model is dynamic. The immanent dimension in the SMC coincides, in the model, with the intersection of the two axes – time and emotion – which produces a 2D and circular life or a “flat life” (see Figure 3). In the flat life, the repetition of experience cycles is determined by the interaction between emotional memories and projective experiences; that is, a circuit that moves between dependence and the need for gratification.

The insertion of the third axis results in a different interpretative capacity (Paoletti and Ben-Soussan, 2019). The idea of aspiration, or intentionality aimed at the pursuit of a predetermined result, enables the implementation of interpretative capacity (Deci, 1971). In the SMC, the level of interpretation is represented in the relation between the center and the periphery. The dimension of the transcendent self is a global relation between the center and the whole space of the sphere.

Similarly, in Wilber’s theory of the self (Wilber, 1979), a higher-order structure is assumed to emerge through a differentiation of the preceding, lower-order level at each stage of psychological development. The emergent structure is considered more complex and, therefore, more unified. The higher-order structure is introduced to consciousness and eventually the self identifies with it. For example, as the body emerged from its fusion with the material world, consciousness became a body-self, identified with the body. As language emerged in awareness, the self began to shift from a solely biological body-self to a syntactical ego and eventually identified itself with language and operated as a syntactical self. While the detection of levels of self based on abstraction ability is similar to Paoletti’s model, the definition of the levels is different. For example, in his model, language is not considered as a distinct level in the development of the self. In addition, the distinctive feature of the more developed self is not greater complexity but rather unitarity. The feature of unitarity is considered a result of development in Paoletti’s model, while in Wilber’s theory, the self seems to be originally intended as a unit able to identify with different “levels, lines, and states” (Wilber, 2000).

**Figure 8:**
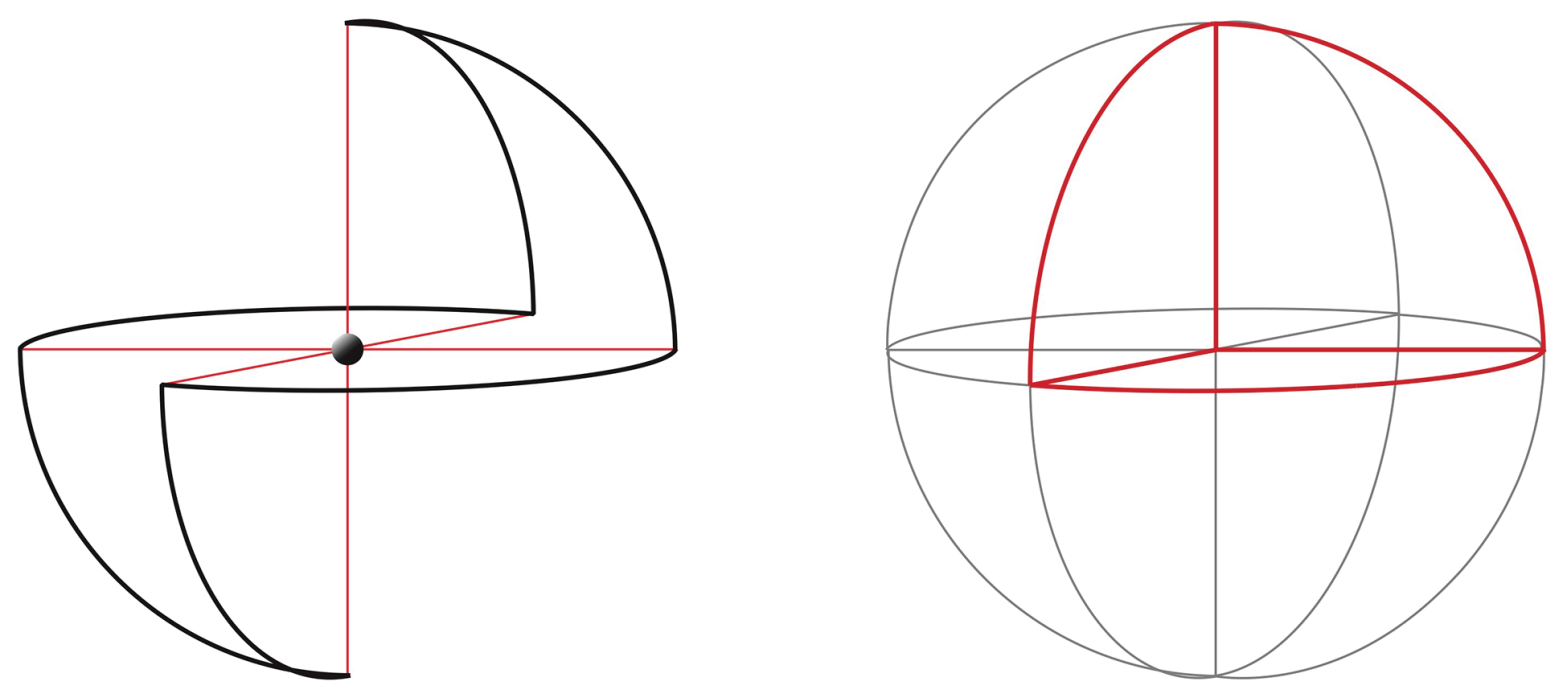
The eight fields of life: body, family, career, friendship, spirituality, relationships, finance, and collectivity. The first four are located in the lower part of the sphere, while their respective counterparts are located in the upper part. Adapted from Paoletti and Selvaggio (2011).

Many models related to the current model are 2D. For example, while Maslow’s hierarchy of needs is a theory of human motivation (Maslow and Lewis, 1987), and not a model of consciousness but of needs, it is highly relevant to the SMC. The SMC is 3D, taking three axes into consideration simultaneously, including basic needs and higher motivations. As previously described (Paoletti and Selvaggio, 2011) in the SMC, the six directions designated by the three axes unfold to eight fields of life, namely body, family, career, friendship, spirituality, relationships, finance, and collectivity. The first four are located in the lower part of the sphere, while their respective counterparts are located in the upper part (Paoletti and Selvaggio, 2011, see Figure 8). Here too, geometry is significant – the lower four are related to more basic needs, while the upper four are related to values (Paoletti, 2005). Similarly, in Maslow’s model, there are at least five sets of goals, namely physiological, safety, love, esteem, and self-actualization, which are proposed to be basic needs that can be simultaneously expressed or satisfied. According to Maslow, the appearance of one need usually rests on the prior satisfaction of another, more pre-potent need. The lower needs in Maslow’s model, which are “instinctive” in nature, are similar to the lower part of the sphere in the SMC.

Thus, in the flat life condition, while we agree with Maslow on many aspects that classifications of motivation should be based also upon conscious goals rather than on drives (Maslow and Lewis, 1987; Paoletti, 2005; Paoletti and Ben-Soussan, 2019). Thus, the transition to a spherical life, which is accompanied by a change of focus from needs to values (Paoletti, 2005), is further accompanied by an intentional shift away from DMN activity and toward clear goal state, represented by the center of the sphere (Paoletti, 2002a,b; Paoletti and Ben-Soussan, 2019; Paoletti et al., 2020).

Similar to the SMC, based on knowledge, intelligence, and learning theories, and especially on Gardner’s theory of multiple intelligences and Maslow’s approach to transcendence, Drigas and Pappas (2017) proposed an eight-layer pyramid of knowledge. In their model, the individual must master each layer in order to reach transcendence, which involves greater freedom from biological and social conditioning. Each increase in level of the pyramid involves a higher state of self-organization, awareness, and consciousness, alongside reduced entropy. Drigas and Pappas define consciousness as a monitoring, regulation, and adaptation process that enables individuals to move from one layer to the next. In this context, the sphere could be regarded as a two converging pyramids or a clepsydra. In this sense, climbing the pyramid can be regarded as moving toward the center of the sphere. Yet, while for Drigas and Pappas, the individual has to conquer all the levels (including stimuli, data, information, knowledge, expertise and discrimination, self-actualization, and universal knowledge) in order to reach transcendence, it should be noted that some traditions, such as the Vedas, claim that self-transcendence and state of one’s essence and being would actually lead to universal – complete knowledge (Alexander et al., 1987). In this sense, Drigas and Pappas’ theory may be different from the SMC, as we distinguish between consciousness and intelligence. In this respect, it could be related to theories of consciousness and information processing, such as Baars and Franklin’s LIDA (Baars and Franklin, 2007), for which specific neural correlates are suggested.

#### Self-Awareness and Silence

According to the SMC, self-awareness is expressed as a relationship between contents placed along the axes and the concentric circles of the selves. The highest degree of self-awareness would be represented as an equal relationship between the periphery of the sphere, or the extremes of the axes, and the center, where we place consciousness-as-such. We assume, in the SMC, that an equal relationship between consciousness and contents can be reached only intentionally. From a subjective, phenomenal point of view, this state of self-awareness could manifest itself as intentionally sought silence, encompassing all the external and internal stimuli. As we have seen above, sensory and perceptual deprivation has been reported as a positive or aversive experience (Hebb, 1955; Feinstein et al., 2018; Ben-Soussan et al., 2019). For example, while isolating participants against their own will can be considered white torture (Brown, 2007; Mausfeld, 2009), over the centuries, people have utilized isolation as means of getting reconnected to themselves and achieve higher states of consciousness (Ustinova, 2009, 2017). Similar to sensory deprivation, silence can be experienced as a positive or negative experience, depending on the interpretation and valance they give to it. For example, teaching the positive aspects of silence (e.g., ranging from silence the classroom to silence during meditation) can provide people the information on the beneficial effects of silence, therefore increasing the probability that they may voluntarily choose to take this path daily, dependent on their current situation. Thus, willingly staying in silence as it occurs in many meditation practices is actually advantageous for mental and physical well-being (Vago and Zeidan, 2016), emphasizing further the importance of education and the third axis.

In the three phases, process proposed by Depraz et al. (2000) of the act of becoming receptive toward experience is characterized by what they call *letting-come*, and what we defined as *being in the waiting* (Ben-Soussan et al., 2014; Paoletti and Ben-Soussan, 2019). Letting-come is defined by Depraz et al. (2000) as a “gesture,” which produces a state of void of content lacking of any immediate discrimination. In this sense, just as silence is not the absence of sound in “listening to silence” meditative practices (Teschner, 1981; Davies and Turner, 2002; Stratton, 2015), the state at the center of the sphere, when it is experienced as the place of pre-existence, would be not characterized by the absence of contents, but by the equidistant presence of all contents, with none prevailing in its relationship with self-perception. Similarly, the axis of time can be regarded as going inward from (1) the narrative (past/future projected DMN) state to (2) awareness of the present moment (Minimal Self), and then (3) beyond time in the Overcoming of the Self (Wittmann, 2015, 2020; Paoletti and Ben Soussan, 2019; Paoletti et al., 2020), which could possibly be accompanied by observing the unfolding of time from one present time to *n* presents.

#### Identification and Absorption: The Importance of the Third Axis

In the context of meditative practice, Minimal Self-related networks are more active in relation to the experience of here and now (Farb et al., 2007; Berkovich-Ohana and Glicksohn, 2017), while those related to the Narrative Self are more active in relation to mental rumination, mind-wandering, and DMN activation (Hamilton et al., 2011; Hasenkamp and Barsalou, 2012; for a review, see Fox et al., 2015). During mind-wandering, the spotlight of attention is narrow and unintentional due to increased engagement with one mental object (Farb et al., 2012; Lutz et al., 2015; Vago and Zeidan, 2016). Similarly, when we identify with only one of the physical, affective, or abstract thought dimensions, or when these dimensions are in conflict, processes of transcendence and integration of information that we see as the basis for both meditative practices and creative abilities do not occur (Horan, 2009). We propose that this state of conflict can be denoted “identification” (Paoletti, 2008; Paoletti and Selvaggio, 2011).

In terms of the SMC, identification is described as an imbalance along one of the axes. Attention is identified, from time to time, with elements of the past or the future, with emotional experiences, or with aspirations or values, but it is usually not divided between these and something else (Paoletti, 2008; Paoletti and Selvaggio, 2011). For example, in a recent study by (van Elk et al., 2019), participants were presented with awe-eliciting, positive and neutral videos, while they were instructed to get fully absorbed in the scenery or to count the number of perspective changes. In line with previous studies using passive or low-demand tasks, it was expected that the absorption condition would be associated with increased activity in regions comprising the DMN. In contrast, the analytical compared to the absorption condition was expected to result in an increased activation of the frontoparietal attention network. In line with their hypothesis, van Elk et al. (2019) found stronger activation in the DMN in the absorption condition. Nevertheless, this was less the case when participants were watching awe videos, suggesting that while watching awe videos, participants were deeply immersed in the videos and that levels of self-reflective thought were reduced. In contrast, the insula and other key regions of the fronto-parietal network were most strongly activated in the analytical condition when participants were watching awe videos compared to positive and neutral videos. In this respect, the sense of self is characterized by the element with which the subject identifies. In the state of identification, there is not a distinct perception of oneself and the element toward which attention is directed. Similarly, in “listening to silence,” attention must not be led away by noise; rather, the practitioner is required to encompass noise in paying attention to silence. Thus, the division of attention between the element and oneself can hypothetically be the path to self-awareness, as defined above: an intentionally sought equal relationship between self-perception (the center of the sphere) and the phenomenal element of experience placed along the axes.

Many meditation techniques involve observing the experiential field by allowing thoughts and sensations to arise and pass without clinging to them (Cahn and Polich, 2006), thus deactivating the DMN (Brewer et al., 2011; Hasenkamp and Barsalou, 2012; Tomasino et al., 2012; Garrison et al., 2015). Study findings further suggest that awareness of subjective or phenomenal aspects of experience in the present moment involves neuronal populations with responses marking transient body states, in particular the somatic and interoceptive insular cortices (Damasio, 1999; Craig, 2004; Critchley et al., 2004; Farb et al., 2007).

Somatic marker or momentary self-awareness areas have also been implicated in OMM or mindfulness-based meditation (Lutz et al., 2008b). Using EEG, Travis et al. (2010) found that cortical midline circuits activated during Transcendental Meditation (TM) practice overlap with those of the DMN (Raichle et al., 2001). Travis et al. concluded that since activation in these default mode brain areas was higher during TM compared to rest (with eyes closed), the experience of contentless thought with continued self-awareness during TM practice could be different from autobiographical or mind-wandering thoughts (Travis, 2014).

As noted above, similar to the SMC’s place of pre-existence in the center of the sphere, several theoretical descriptions (Vago and Zeidan, 2016) and empirical investigations (Josipovic, 2014; Vieten et al., 2018) of subjective experiences of non-duality (a sense of oneness or a perceived dissolution of the distinction between the observer and the contents of observation) have emerged in recent years. These states are thought to occur when the silent background awareness encountered in meditation becomes sufficiently stabilized and integrated with the daily waking experience, so that the habitual reified dualities between subject and object, self and other, and in-group and out-group dissipate. It is hypothesized that these states lead to a more spacious and compassionate way of being (Josipovic, 2016).

Identification has rarely been examined, as it is very elusive: the moment you voluntarily pay attention to yourself (thus hypothetically activating the third axis), you are no longer completely identified. Thus, while both identification and absorption are focused on a specific perceptual, imaginative, or ideational experience, perhaps the awareness axis gives the absorption experience its three-dimensionality. Taking pain as an example, we consider what happens when we are identified, and when we voluntarily add an additional perspective.

Pain is a conscious experience that can be considered an interpretation of nociceptive input influenced by memories, as well as emotional, pathological, genetic, and cognitive factors. It is thus more than just a qualitative experience or set of experiences (Mordeniz, 2016). Beyond its immediate unpleasantness, emotions experienced in the anticipation of pain and in response to its meanings and perceived future consequences are related to the DMN and salience network (Kucyi et al., 2014; van Ettinger-Veenstra et al., 2019). The overlap between the patterns of cerebral activity associated with feelings of pain, emotions, and motivational states in the ACC and insula is consistent with their contribution to basic aspects of self-representation, self-regulation, and consciousness (Wiech et al., 2008). Salomons et al. (2004) showed that perceived control over pain decreased pain-related responses in the ACC and insula. In accordance, the placebo effect, which is closely related to self-determination and interpretation of stimuli, has been found to decrease pain intensity and cerebral responses to pain in brain areas including the ACC and insula (Petrovic et al., 2002; Wager et al., 2004; Bingel et al., 2006; Wiech et al., 2008). In addition, the insula and adjacent areas are activated when individuals view or become aware of the delight, pain, or disgust of others, as when they experience these emotions first-hand, and this activation is modulated by individual empathic tendencies (Agnati et al., 2013).

In this context, it is important to note that the brain network governing meditation has been studied using a variety of meditation practices and techniques eliciting different mental processes, such as silence and attention to one’s body and empathy, which are all linked to increased insular activity (for a review, see Tomasino et al., 2012). Recent studies have also shown that meditation inhibits or relieves pain and reduces pain-related neural activity in the ACC, insula, secondary somatosensory cortex, and thalamus (Nakata et al., 2014). It was further found that expert meditators, as compared to novices, report equal pain intensity but less unpleasantness. This difference was associated with enhanced activity in the insula and the anterior mid-cingulate among experts, while experiencing pain (Lutz et al., 2013). The authors have suggested that cultivating experiential openness downregulates anticipatory representation of aversive events and increases the recruitment of attentional resources during pain, which is associated with faster neural habituation.

Cognitive modulations of pain are related to activation of prefrontal brain areas (DLPFC, VLPFC, and ACC), which modulate activation in pain-associated regions in the cortex (ACC, SI, SII/insula, and thalamus). Affect labeling, or putting feelings into words, has long been thought to help manage negative emotional experiences. Relative to other forms of encoding, affect labeling diminishes the response of the amygdala and other limbic regions and produces increased activity in the right ventrolateral prefrontal cortex (RVLPFC), a region associated with the symbolic processing of emotional information and with top-down inhibitory processes (Lieberman et al., 2007; Burklund et al., 2014). Together, these results suggest that putting feelings into words might activate the RVLPFC, which in turn might dampen the response of the amygdala, thus helping to alleviate emotional distress. These results suggest that detaching one’s self from being identified completely in a specific situation (e.g., anxiety about current situation, emotional or physical pain), through intentional affect labeling, can diminish emotional reactivity.

In addition, while many mediation practices decrease DMN activation, as noted above, nondirective meditation, which permits mind-wandering, involves extensive activation of brain areas associated with episodic memories and emotional processing, than during concentrative practice or rest conditions (Xu et al., 2017). In relation to this, one should keep in mind that the DMN is further related to many positive attributes of consciousness and cognition, such as creativity and imagination (Agnati et al., 2013; Berkovich-Ohana and Glicksohn, 2017).

## Silence, Divided Attention, and Regeneration of Memories

We now return to silence as a means of reaching the state of consciousness without contents, or, perhaps more accurately, “with all contents.” Scholl et al. (2010) have shown that a specific set of neurons is dedicated to silence, distinct from those that deal with sound. Silence cannot, therefore, be conceptualized solely as a cancellation or deletion of sound. This coincides with a fundamental principle of meditative technique, requiring practitioners to move away from rather than eliminate internal chatter, in order to move toward a non-sound, or silence. The effort of moving away from internal chatter while moving toward silence can be seen as a voluntary effort (Tomasino et al., 2012) to divide attention (Meck and MacDonald, 2007; Paoletti and Selvaggio, 2011; Wyart et al., 2015).

Silence also stimulates processes of neurogenesis in the hippocampus, which have in turn been tied to regeneration of memories and creation of new associations (Kirste et al., 2015). Silence can, therefore, be seen as the entrance into a state of neutrality with respect to stressors, stimuli or previous memories. The stressors do not disappear: silence is not the absence of stimuli, but a greater space that allows inner distance and thus better management of stimuli. In this sense, silence represents a state of preparation for emptiness (Teschner, 1981; Davies and Turner, 2002; Stratton, 2015). The state of emptiness is an indefinitely larger space, which can be reached through sensory saturation (Scharfetter, 1981; Vollenweider, 1998; Vollenwieder and Geyer, 2001; Jamieson, 2007). Thus, emptiness is not the absence of references but the equidistant presence of all references, which can be better managed in a wider space.

Self-report and other psychological studies have shown that meditative practice induces increased awareness of perception and body awareness. However, phenomenological studies indicate that meditative practices lead to the experience of complete absence of an essential self or a total Overcoming of the Self. This state might coincide with the central void of the sphere discussed above, and therefore, with consciousness without content. Much is still to be discussed and examined from neuronal and physiological viewpoints, uniting different terminologies, ranging from the possible connection between silence, transcendence, and absorption to the differential effects of identification on the three axes and their neuronal correlates.

## Silence and Consciousness Without Content

We conceptualized silence in meditation as a means of sensory saturation thought to induce experiences of consciousness without content, or silent consciousness (Baars, 2013). We assume that this state can be achieved in meditative practices that provide for sensory expansion. Such is the indefinite expansion of the senses, as described in Patanjali’s Yoga Sutra with the concept of *pratyahara*. According to some, it indicates a kind of coercive control over the senses, but it can be understood instead as training in detachment from sensory perception, or non-attachment to the object of perception, which differs from its inhibition. This detachment would coincide with the practice of widespread attention, the “letting-come” of Depraz et al. (2000), the physiological correlates of which can be viewed as widespread endogenous attention in the open field (Raffone and Srinivasan, 2009; Srinivasan et al., 2009), leading to a form of sensory saturation subserved by the insula (Ben-Soussan et al., 2019; Paoletti and Ben-Soussan, 2019).

It is possible that these experiences of self-transcendence, defined by some as the extent to which individuals conceive themselves as integral parts of the universe as a whole, induce a transformation from a body/ego-based self-identity to a world/universe-centered experience of self, not limited to the self-narrative of the individual practitioner (Vieten et al., 2018). During peak states and deep meditative states, the self becomes one with the universe and experiences positive self-dissolution, the feeling of unity of the three time perspectives (or “timelessness”; Vaitl et al., 2005; Studerus et al., 2011; Wittmann, 2015).

## Concluding Remarks and Future Perspectives

In the current hypothesis paper, we offered a synopsis of data suggesting a mechanism by which silence might enhance change in consciousness. Some of the key regions active in compassion meditation, including the insula, are involved in developing stronger than usual self and agency. Thus, combining knowledge regarding the importance of the insula in emotional modulation, embodiment, and neuroesthetics (Di Dio et al., 2007), with recent studies on time, space, and the insula (Wittmann, 2015; Ben-Soussan et al., 2019), we suggest that being immersed in silence, similarly to being immersed in a state of absorption, can activate the insula (among other areas). This will induce a shift toward the center of the sphere, which will in turn lead to alterations in the perception of time (Glicksohn et al., 2017), sense of unity, and non-duality (Wittmann, 2015), and eventually to enhanced empathy (Lutz et al., 2008a; Singer et al., 2009), which are reported in many contemplative techniques. Future research should examine the SMC with respect to different practices using both neuronal correlates of the different states they produce, as well as some physiological markers, such as heart rate variability and other parasympathetic measures (Travis and Wallace, 1999; Travis and Pearson, 2000), as well as in relation to augmented and virtual reality (Kiourexidou et al., 2015). In addition, similarly to sensory deprivation, which has been found to be beneficial for memory functions, creativity, perception and signal detection, social cognition, and the readiness to change one’s attitudes on social phenomena inducing increased motivation to change critical and maladaptive behavior patterns (for a review, see Vaitl et al., 2005); the importance of voluntarily entering into a state of silence, as an educational conceptual space, can and should be studied also in educational settings, in relation to the functions mentions above, as well as logics and mathematics (Stefaneas and Vandoulakis, 2014).

In the current paper, we focused mostly on the difference between intentional removal of content through an internal mental action (e.g., meditation) versus externally depriving (or saturating) perception. Future studies should also examine the differential theoretical and empirical effects of visual, auditory, and kinesthetic stimulation (such as photic stimulation and yantras, binaural beat, massage, and movement meditation, respectively), and their possible role in shifting position within the SMC (Paoletti et al., 2020). Finally, in line with Maslow’s claim that for some people, one level is more important than others (e.g., self-esteem more than love; Maslow and Lewis, 1987), future studies should examine the relationship between location inside the sphere, personality, and the main fields visited inside the sphere (career, family, and friends). This could be particularly interesting when taking into consideration personality traits such as introversion and extraversion – which are closely related to silence (Cassidy and MacDonald, 2007; Cain, 2013) – and their neural correlates (Stenberg, 1992; Leshem et al., 2019).

## Author Contributions

PP is the creator of the Sphere Model of Consciousness and contributed the parts related to the neuopsychological applications of his model (e.g., place of pre-existence) and the importance of silence in them. TB-S contributed the parts mostly related to neural correlates and the differentiation between absorption and identification. All authors contributed to the article and approved the submitted version.

### Conflict of Interest

The authors declare that the research was conducted in the absence of any commercial or financial relationships that could be construed as a potential conflict of interest.

## Footnotes

^1^Bernard Baars’ GWT (Baars, 1983, 1998, 2002; Baars et al., 2003) is one of the most influential theories of human consciousness. According to this view, contents are categorized in conscious and unconscious perceptions. Conscious perception which gives access to brain resources, in terms of wide broadcasting of information in brain areas. In contrast, unconscious perception involves brain sources in an unconnected or modular way. According to the GWT, consciousness provides a gateway to unconscious knowledge resources in the brain. To exemplify the functional properties of the GW, Baars (1998) used a theater metaphor, referring to it as a useful first approximation (Baars and Franklin, 2007).

^2^Alpha coherence is an electrophysiological measure of functional connectivity, characterized by synchrony relationships between the activities of two neuronal populations. Studies further report higher frontal alpha coherence, slower breath rate, lower sympathetic activity, and higher parasympathetic activity during transcending, compared to periods of undirected thoughts (Travis, 2001; Travis et al., 2002). Enhanced alpha coherence was further found to be related to both increased cognitive flexibility and fine motor control and performance (Babiloni et al., 2011; Ben-Soussan et al., 2013).

^3^It is important to note that the alpha activity remained enhanced in the Ganzfeld despite no indication of diminished brain arousal (Miskovic et al., 2019).
